# Supplementary figures and images for: A type VII secretion system in Group B Streptococcus mediates cytotoxicity and virulence
Source: PLoS Pathog. 2021 Dec 6;17(12):e1010121. doi: 10.1371/journal.ppat.1010121 (PMC8675928; doi:10.1371/journal.ppat.1010121)

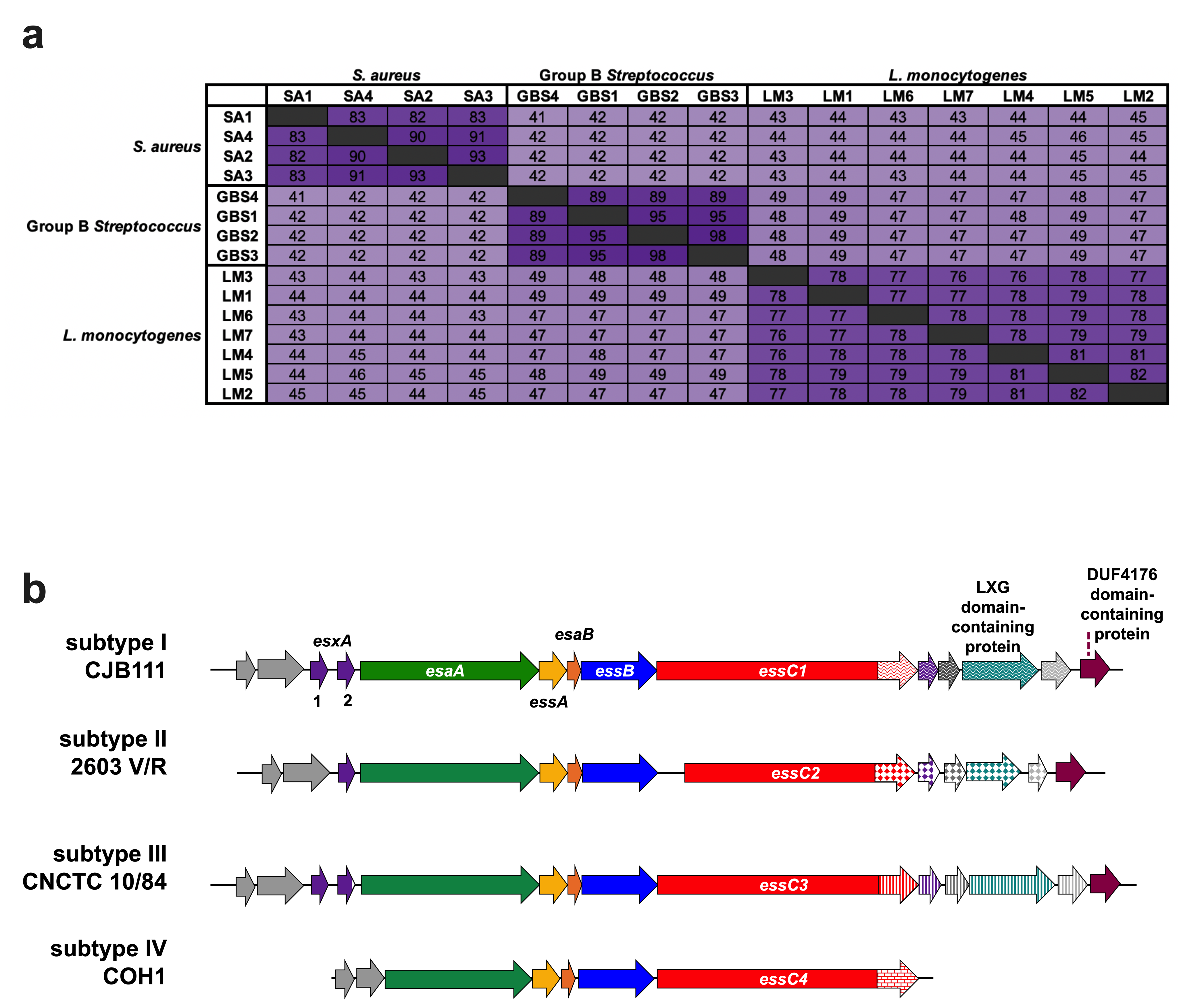

Supplement: S1 Fig — a) Percent identity matrix of full EssC amino acid sequences from example GBS, S. aureus, and L. monocytogenes strains from various T7SS subtypes. Strain information is listed in S2 Table. The purple shading corresponds to the level of identity between two strains (on a spectrum of 0 to 100% identity), with darker shading indicative of higher percent identity. b) Diagram of putative T7SS loci from example strains across GBS T7SS subtypes I—IV. CJB111 (accession CP063198.2) is an example of subtype I and expresses the EssC1 variant (ID870_04200). 2603 V/R (accession NC_004116.1) is an example of subtype II and expresses the EssC2 variant (SAG_RS07895). CNTC 10/84 (accession NZ_CP006910.1) is an example of subtype III and expresses the EssC3 variant (W903_RS05455). COH1 (accession NZ_HG939456.1) is an example of subtype IV and expresses the EssC4 variant (GBSCOH1_RS05095). Genes in purple encode WXG100 or WXG100-like proteins, gene in teal encodes a LXG domain-containing protein, and gene in maroon encodes a DUF4176 domain-containing protein. Genes in various shades of gray are either annotated as hypothetical or do not have a predicted function. Arrows with patterns indicate T7SS subtype-specific genes that exhibit little to no homology to those present in other GBS T7SS subtypes. Putative core genes of the operon are esaA through essC. (TIF) [file ppat.1010121.s005.tif]

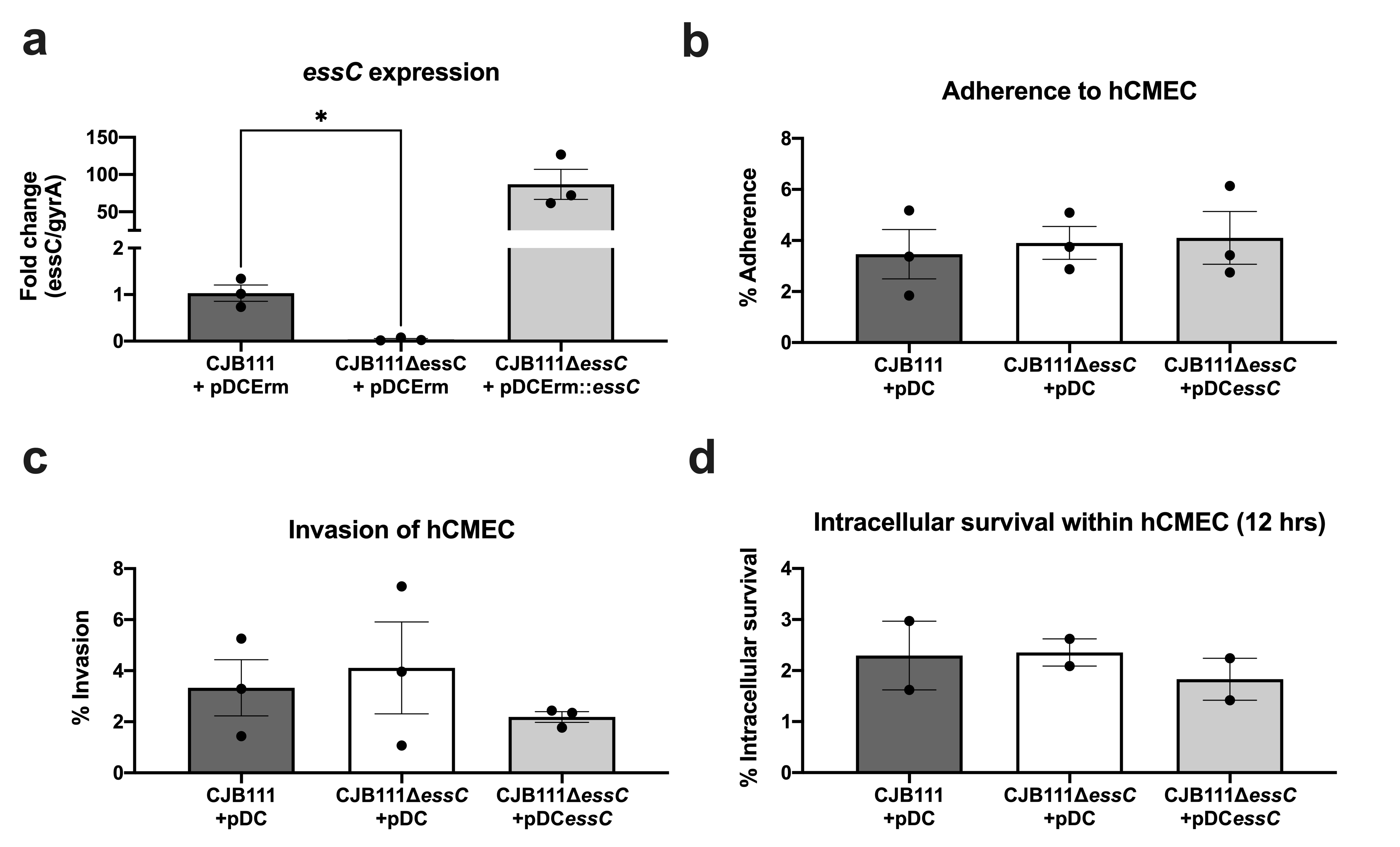

Supplement: S2 Fig — a) Expression of essC in CJB111+ pDC, CJB111ΔessC+ pDC, CJB111ΔessC + pDCessC strains by qRT-PCR. T7SS gene expression was normalized to housekeeping gene gyrA. Statistics reflect the repeated measures, one way ANOVA with Dunnett’s multiple comparisons test to CJB111ΔessC+pDC, p < 0.05, *. Data indicate the mean of three independent experiments and error bars represent standard error of the mean. The CJB111+ pDC, CJB111ΔessC+ pDC, CJB111ΔessC + pDCessC strains were further evaluated for b) adherence to (n = 3), c) invasion of (n = 3), or d) intracellular survival (12 hrs; n = 2) in human cerebral microvascular endothelial cells (hCMEC). Data represent percent CFU recovered of the initial inoculum and were performed in technical duplicates or triplicates. (TIF) [file ppat.1010121.s006.tif]

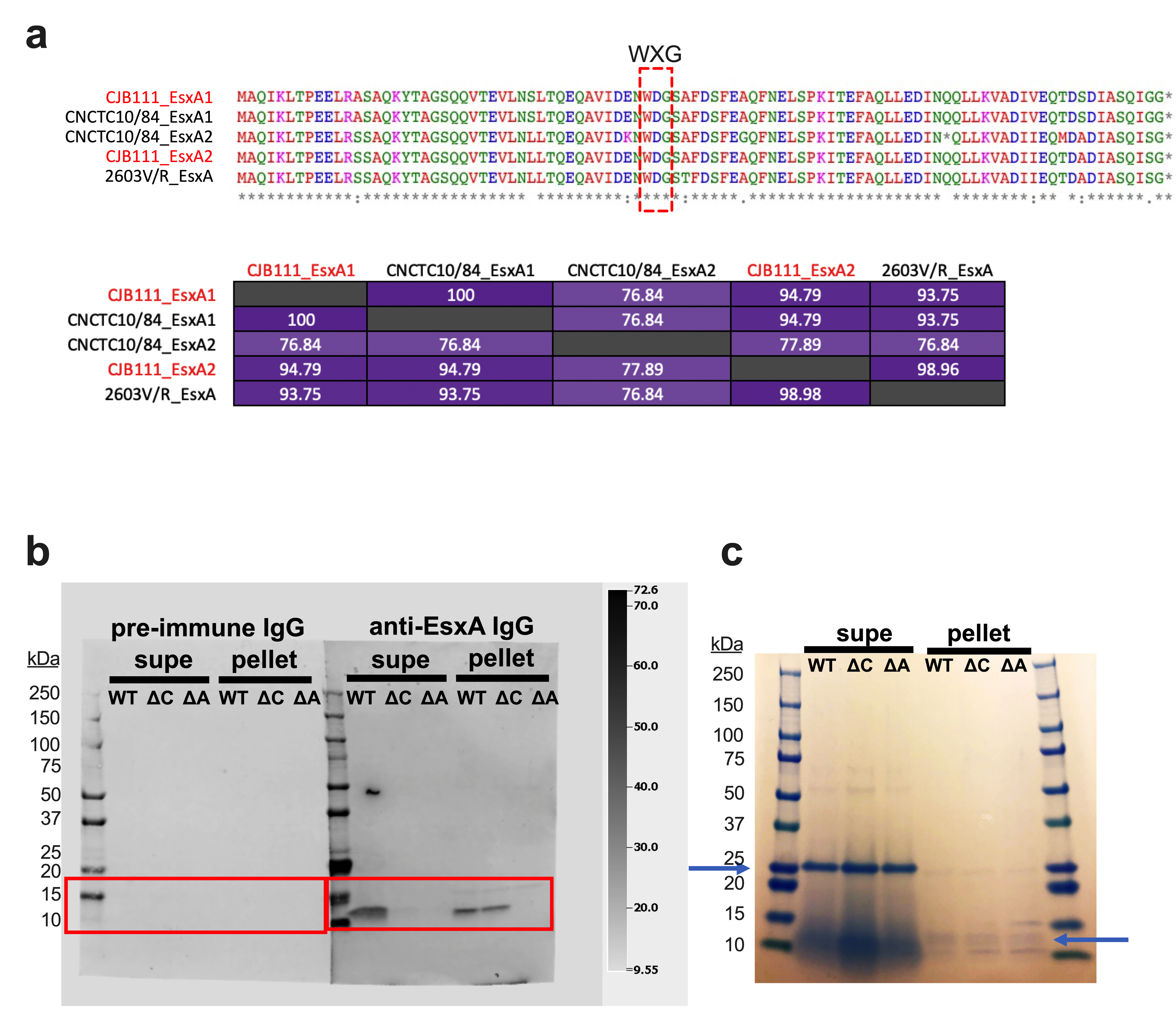

Supplement: S3 Fig — a) ClustalW alignments and percent identity matrix of EsxA amino acid sequences from GBS T7SS subtypes I-III that are encoded upstream of the putative GBS T7SS loci. Subtype I example strain CJB111 (accession: NZ_CP063198.2) encodes EsxA1 and EsxA2. Subtype II example strain 2603V/R (accession: NC_004116.1) encodes EsxA2. Subtype III example strain CNCTC 10/84 (accession: NZ_CP006910.1) encodes EsxA1 and a truncated EsxA2. In the above matrix, the purple shading corresponds to the level of identity between two strains (on a spectrum of 0 to 100% identity), with darker shading indicative of higher percent identity. b) un-cropped, full picture of the Western blot shown in Fig 4B. The only other band (CJB111 supernatant) may indicate oligomerization of monomeric EsxA over time, but this needs to be further investigated. c) Coomassie-stained SDS PAGE gel indicating that wells were equally loaded (see blue arrows) for the Western blot shown in Fig 4B. (TIF) [file ppat.1010121.s007.tif]

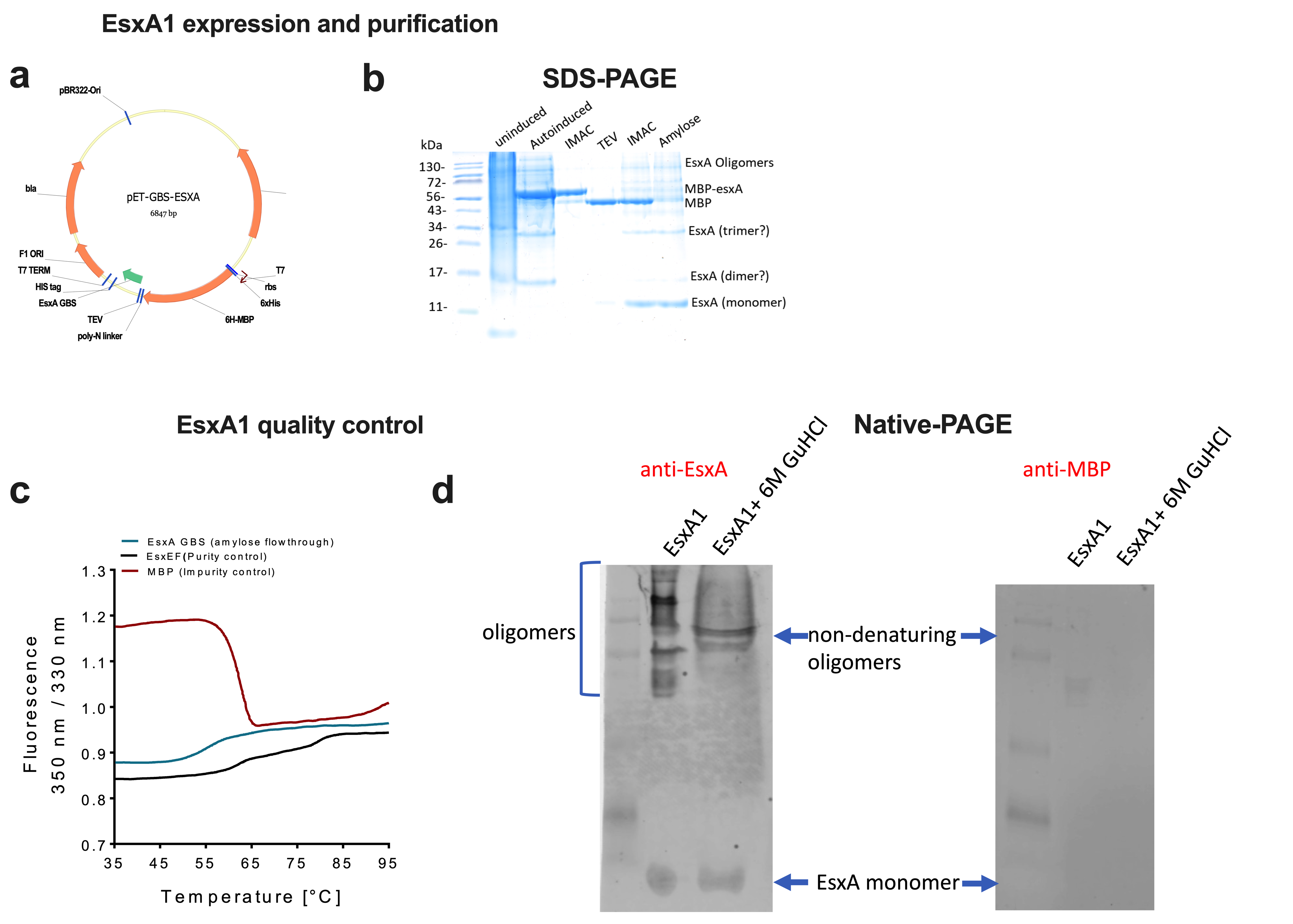

Supplement: S4 Fig — a) Plasmid map of CJB111 esxA1 cloned into the backbone of pET vector pML3339. EsxA1 expressed from this vector is 6xhisMBP-tagged to facilitate nickel affinity and amylose affinity column purification. b) SDS-PAGE gel of EsxA1 during purification: un-induced BL21 culture, auto-induced BL21 culture, post-nickel affinity column (IMAC), post-TEV cleavage/dialysis; post amylose column to remove cleaved MBP, final EsxA1. Final EsxA1 product shows a ~11 kDa monomer as well as higher order oligomers. c) Quality control of the purified EsxA1 by differential scanning fluorimetry (Tycho, NanoTemper Technologies). Maltose binding protein was run as a negative control and mycobacterial EsxEF was run as a positive control. d) Native-PAGE indicating that most EsxA1 oligomers resolve to the monomeric state upon treatment of protein with 6M guanidine HCl for 30 minutes at room temperature. EsxA1 bands stain with anti-EsxA1 rabbit antiserum but not with anti-MBP antibody. (TIF) [file ppat.1010121.s008.tif]

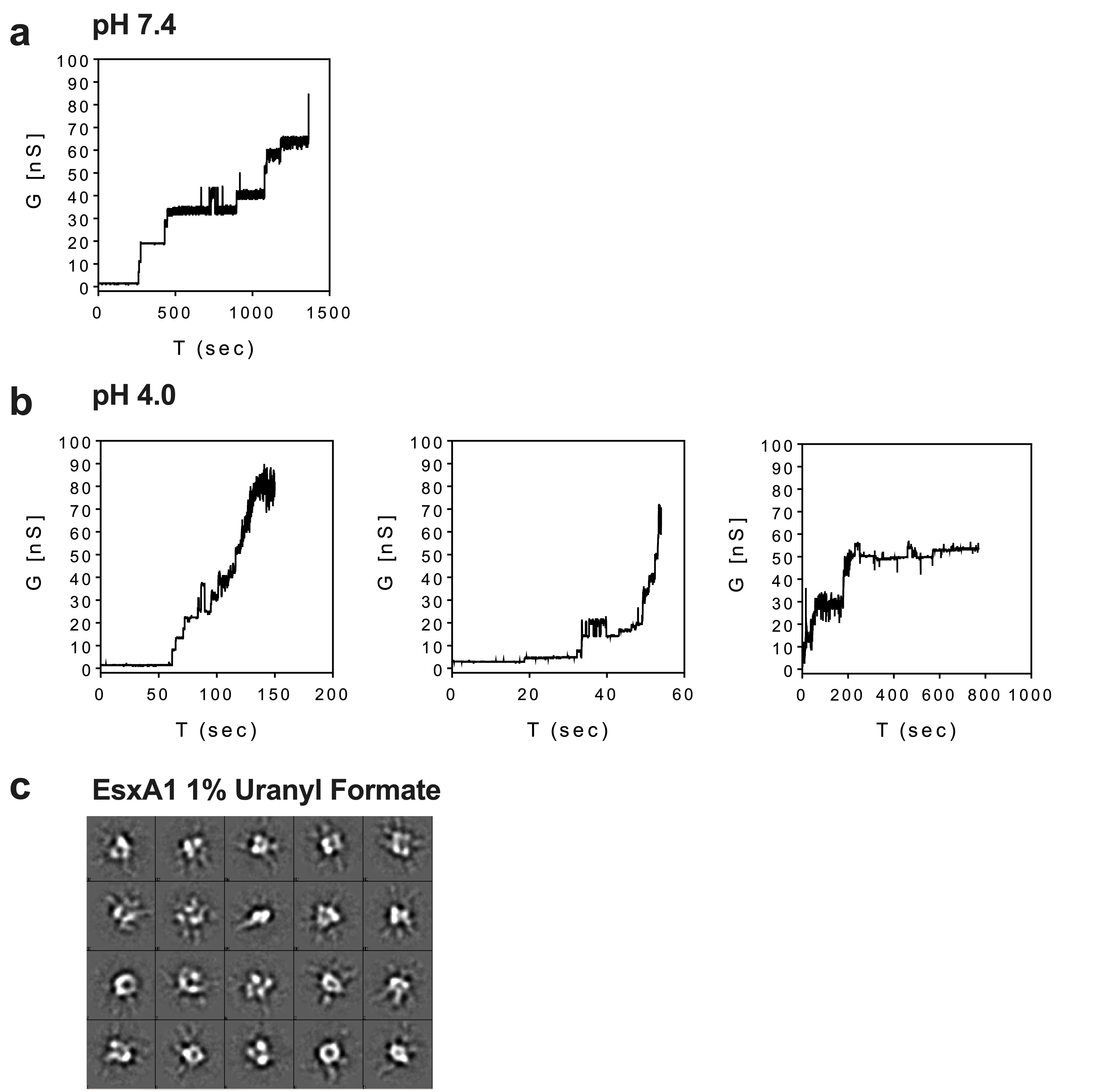

Supplement: S5 Fig — Additional current traces of recombinant CJB111 EsxA1 pore formation in DphpC lipid bilayers at a) pH 7.4 and b) pH 4.0 in 25 mM sodium phosphate 1M KCl. c) Additional reference-free 2D class averages of negatively stained EsxA1 imaged by transmission electron microscopy. (TIF) [file ppat.1010121.s009.tif]
